# Utility of Restaging MRI Following Neoadjuvant Chemoradiotherapy for Stage II-III Rectal Adenocarcinoma

**DOI:** 10.7759/cureus.19037

**Published:** 2021-10-25

**Authors:** Mahmoud Aryan, Thomas Read, Lindsey Goldstein, Nathan Burriss, Joseph R Grajo, Patricia Moser, Thomas J George, Sanda Tan, Atif Iqbal

**Affiliations:** 1 Department of Surgery, University of Florida College of Medicine, Gainesville, USA; 2 Department of Radiology, University of Florida College of Medicine, Gainesville, USA; 3 Department of Hematology and Oncology, University of Florida College of Medicine, Gainesville, USA

**Keywords:** neoadjuvant, chemoradiation, mri, cancer, rectal

## Abstract

Background

Magnetic resonance imaging (MRI) is currently utilized for the pretreatment staging of locally advanced rectal cancer; however, there is no consensus regarding the utility of repeat MRI for restaging following neoadjuvant chemoradiotherapy (CRT). In this study, we aimed to investigate the clinical utility of restaging MRI after CRT in patients with clinical stage II-III rectal cancer.

Methodology

We performed a retrospective observational study at a tertiary care hospital. Our study population included patients with clinical stage II-III rectal cancer treated with neoadjuvant CRT who underwent both pre- and post-CRT MRI followed by surgical resection from 2012 to 2017. MRIs were reviewed by radiologists with an interest in rectal cancer MRI imaging using a standardized template. The utility of post-CRT MRI was evaluated by assessing its impact on change in surgical planning, concordance with pathologic staging, and prediction of surgical margins.

Results

A total of 30 patients were included in the study; 67% had clinical stage III and 33% had stage II disease based on pre-CRT MRI. Post-CRT MRI findings did not lead to a change in the originally outlined surgical plan in any patient. Compared to pre-CRT MRI, post-CRT MRI was not significantly more accurate in predicting T stage (k = 0.483), N stage (k = 0.268), or positive surgical margins (k = 0.839).

Conclusions

Due to poor concordance with pathologic staging, inability to more accurately predict surgical margin status and the absence of a demonstrable change in surgical treatment, post-CRT restaging with MRI, in its current form, appears to be of limited clinical utility.

## Introduction

Magnetic resonance imaging (MRI) is widely utilized for the staging of locally advanced rectal adenocarcinoma [1-3]. Patients found to have clinical stage II-III cancers are often treated with chemoradiotherapy (CRT), followed by surgical resection [4,5].

The utility of pre-CRT MRI is widely accepted based on its ability to clinically stage the tumor, guide therapy, assess the involvement of adjacent structures and threatened margins, and determine the extent of surgical resection [1,4]. However, the utility of post-CRT MRI is not well established. Moreover, concerns over the accuracy of post-CRT MRI exist [6]. In this study, we aimed to investigate the clinical utility of restaging MRI after CRT in patients with clinical stage II-III rectal cancer.

## Materials and methods

**Figure 1:**
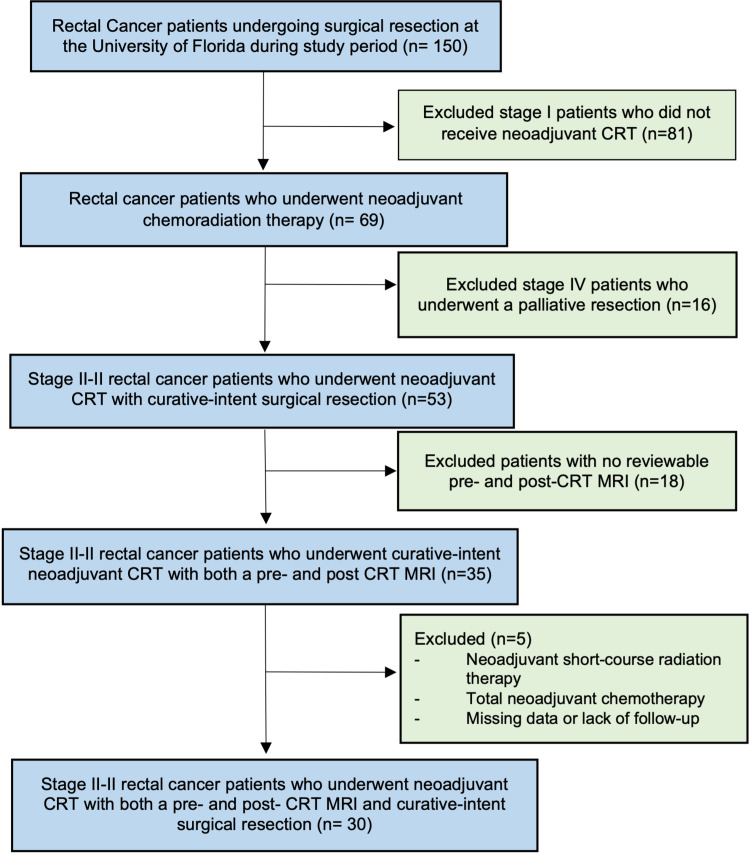
Inclusion diagram. CRT: chemoradiotherapy; MRI: magnetic resonance imaging

We performed a retrospective review of consecutive patients undergoing surgery for rectal adenocarcinoma at the University of Florida Health between March 2012 and March 2017, which was approved by the institutional review board of the University of Florida. The study population included patients with clinical stage II-III rectal cancer treated with neoadjuvant CRT followed by proctectomy, who underwent both pre- and post-CRT MRI. We excluded clinical stage I patients who did not receive CRT, stage IV patients who underwent a palliative resection, those with a lack of a reviewable pre- and post-CRT MRI, and those with missing data or lack of follow-up. To avoid variability, patients who underwent neoadjuvant short-course radiation therapy or those who underwent total neoadjuvant therapy with long-course chemotherapy were excluded. A detailed inclusion diagram is shown in Figure 1.

Data on the medications used for chemotherapy and the time period of administration were recorded to represent the various drug regimens. To account for the impact of time interval on staging results, the time periods from pre-CRT MRI to the start of neoadjuvant therapy, the end of neoadjuvant therapy to post-CRT MRI, and post-CRT MRI to surgery were recorded in weeks.

All post-CRT MRIs (100%) and nearly all pre-CRT MRIs (90%) were performed at the University of Florida Health under the routine Rectal Cancer Protocol in either a 1.5T Siemens Avanto or 3T Siemens Verio MRI machine (Siemens, Munich, Germany). All of these scans included a small field-of-view, high-resolution axial, sagittal, and coronal T2-weighted images along with post-contrast axial, sagittal, and coronal T1-weighted images. Additionally, these scans routinely included axial diffusion-weighted imaging and were initially read by a radiologist who was specialized in abdominal and rectal cancer imaging. Although all of our post-CRT MRIs (100%) were performed at the University of Florida Health, three of our pre-CRT MRIs (10%) were retrieved from outside institutions. To account for any variability in interpretation and reporting over time, MRI results were re-reviewed by two subspecialized radiologists with formal rectal MRI training participating in this study. A standardized University of Florida Health Rectal Cancer template was utilized for this review. The MRI template included all the required elements for rectal cancer MRI, as defined in the “Cancer Care Ontario” template and required by the National Accreditation Program for Rectal Cancer (NAPRC) by the American College of Surgeons [7,8]. Surgical resection was performed between six and twelve weeks from neoadjuvant therapy. Surgical pathology data were recorded via a standardized template, which comprised all elements included in the College of American Pathologists’ Cancer Protocol for rectal cancer, as recommended by the NAPRC [8].

Utilizing the REDcap data collection system, descriptive variables related to patient demographics were identified. Outcome variables including the patient’s operative data, templated MRI, and surgical pathology reports were also recorded. The utility of post-CRT MRI was evaluated by assessing its impact on change in surgical planning, concordance with pathologic staging, and prediction of positive surgical margins.

All patients were presented in a multidisciplinary tumor board, notes of which were reviewed to determine the role of the post-CRT MRI in changing the surgical plan. The planned operation was recorded prospectively during the tumor board meetings. These notes were documented by the gastrointestinal oncology nurse navigator at each tumor board meeting and entered into the patient’s chart. This documentation was then compared to the operative report.

Statistical analysis was conducted using SPSS version 21 (IBM Corp., Armonk, NY, USA). Quantitative data were compared using mean measurements. Variables were compared by exact chi-square test or independent samples t-test for categorical and continuous variables, respectively. Pre- and post-CRT MRI findings were correlated with pathologic staging using concordance percentages, as well as weighted kappa (κ) inter-rater statistical agreement. A κ of <0.5 implied an absence of consistent agreement, whereas a κ of >0.5 pointed toward the presence of an appreciable correlation between MRI and pathologic staging.

## Results

Of the 150 rectal cancer patients identified over the five-year period, 30 were included in the final analysis. Patients were excluded for stage I disease not receiving CRT (n = 81), stage IV patients undergoing palliative resection (n = 16), lack of a reviewable pre- and post-CRT MRI (n = 18), and missing data or lack of follow-up (n = 5).

Patient age ranged from 31 to 79 years, with 15 (50%) males and an average body mass index (BMI) of 27 kg/m^2^. Comorbidities within our cohort included coronary artery disease (13%), chronic obstructive pulmonary disease (6.6%), cirrhosis (3.3%), and diabetes (10%). The American Society of Anesthesia (ASA) classification prior to surgery included class II (77%), class III (20%), and class IV (3%). The remainder of the baseline characteristics of our population are outlined in Table 1. Pre-CRT MRI staged 33% as clinical stage II and 67% as stage III disease. The tumor board review resulted in a change in the clinical stage in six patients (upstaged in three and downstaged in three), primarily due to MRI re-interpretation by an expert radiologist on the tumor board. All patients underwent transabdominal resection (low anterior resection 87%, abdominoperineal resection 13%). Positive surgical margins (tumor ≤1 mm of margin) were noted in 17% of patients, 80% of whom had evidence of “threatened mesorectal margins” on pre-CRT and post-CRT MRIs.

**Table 1:** Baseline Characteristics BMI: body mass index; CAD: coronary artery disease; COPD: chronic obstructive pulmonary disease; ASA: American Society of Anesthesia; LAR: low anterior resection; APR: abdominoperineal resection

Baseline characteristic		n (%)
Age, mean years (SD)		60 (10)
Gender, n (%)	Male	15 (50%)
	Female	15 (50%)
BMI, mean kg/m^2^ (SD)		27 (5.1)
Steroid use, n (%)		18 (60%)
ASA class, n (%)	I	0
	II	23 (77%)
	III	6 (20%)
	IV	1 (3%)
Comorbidities, n (%)	CAD	4 (13%)
	COPD	2 (6.6%)
	Cirrhosis	1 (3.3%)
	Diabetes	3 (10%)
Charlson Comorbidity Index, mean (range)		4.4 (2–11)
Adjuvant chemotherapy, n (%)		22 (73%)
Clinical T stage, n (%)	T1	0%
	T2	7%
	T3	73%
	T4	20%
Clinical N stage, n (%)	N0	20%
	N1	47%
	N2	33%
Year of surgery, n (%)	2013	1 (3%)
	2014	2 (7%)
	2015	8 (27%)
	2016	13 (43%)
	2017	6 (20%)
Procedure, n (%)	LAR	26 (87%)
	APR	4 (13%)
Surgical approach, n (%)	Laparoscopic	17 (55%)
	Robotic	13 (42%)

Patients started neoadjuvant therapy at a mean interval of 3.3 ± 1.9 weeks following pre-CRT MRI. The mean duration of chemotherapy was 5.9 ± 2.5 weeks. The type of chemotherapy regimens used are represented in Table 2, the majority being capecitabine (57%), with the other regimens including continuous intravenous-infusion (CIVI) fluorouracil (5-FU) (17%), 5-FU (17%), combination folinic acid + fluoro­uracil + oxali­platin (6%), and combination capecitabine + 5-FU (3%). The mean duration between the end of neoadjuvant therapy to the post-CRT MRI was 4.5 ± 2.2 weeks, whereas patients underwent surgery at an average of 3.9 ± 1.9 weeks following post-CRT MRI.

**Table 2:** Chemotherapy regimens. CIVI: continuous intravenous-infusion; 5-FU: fluorouracil; FOLFOX: folinic acid + fluoro­uracil + oxali­platin

Chemotherapy	Number of patients
Capecitabine	17 (57%)
CIVI 5-FU	5 (17%)
5-FU	5 (17%)
FOLFOX	2 (6%)
Capecitabine and 5-FU	1 (3%)

**Figure 2:**
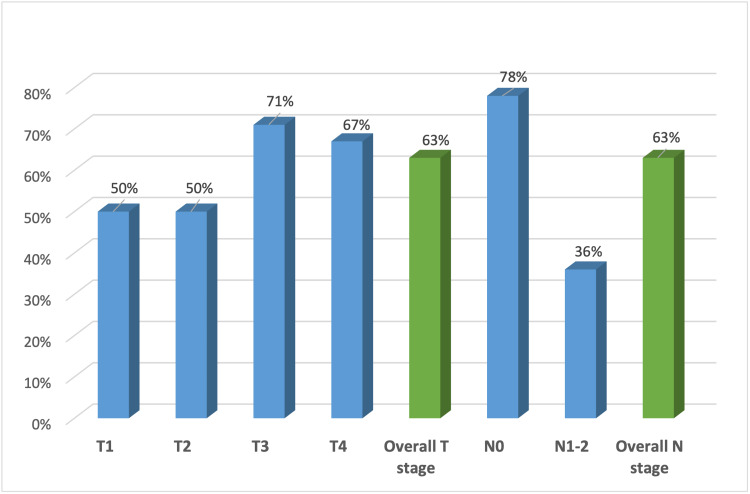
Concordance between post-CRT MRI and pathologic staging. CRT: chemoradiotherapy; MRI: magnetic resonance imaging

The impact of post-CRT MRI on clinical care was then assessed. Although the post-CRT MRI showed downstaging of both the T stage (23%) and N stage (67%), it did not lead to a change in the originally outlined surgical plan in any patient. To determine the concordance of MRI and pathologic findings, concordance values were calculated for both pre- and post-CRT MRI. The overall concordance of pre-CRT MRI with pathologic staging for all patients was 57% (T stage) and 40% (N stage), while that of post-CRT MRI was 63% (T stage) and 63% (N stage) (Figure 2). Although all patients (100%) who were found to have “threatened” margins on MRI had positive surgical margins, the pre- and post-CRT MRIs equally predicted such margins. The negative predictive value for an MRI demonstrating a “threatened margin” was lower than the positive predictive value of this measure. Moreover, the post-CRT MRI could not accurately predict the T stage (κ = 0.483) or N stage (κ = 0.286) and did not add value over the pre-CRT MRI in predicting positive surgical margins (κ = 0.839), as shown in Table 3.

**Table 3:** MRI prediction of pathologic stage and surgical margins. κ = kappa; CRT: chemoradiotherapy; MRI: magnetic resonance imaging

	Change in the originally outlined surgical plan	Pathologic stage prediction		Positive surgical margin prediction
		T stage	N stage	
Pre-CRT MRI, Concordance % (κ)	-	57% (0.349)	40% (0.188)	97% (0.839)
Post-CRT MRI, Concordance % (κ)	0%	63% (0.483)	63% (0.268)	97% (0.839)

## Discussion

Our data challenge the practice of routine restaging with standard anatomic pulse-sequence MRI after neoadjuvant CRT for patients with clinical stage II-III rectal cancer. Post-CRT MRI findings did not alter the predetermined surgical plan in any of our patients. Post-CRT MRI staging, when performed at the time interval used in our study, also failed to add further accuracy in predicting the pathologic stage or margin status compared to pre-CRT MRI. Our data also highlight the utility of multidisciplinary tumor boards.

Our data are consistent with several previous reports that allude to the inaccuracy of MRI for restaging rectal cancer following CRT [9-11]. Restaging with MRI following CRT is challenging due to various reasons such as differentiating fibrosis from fibrotic residual tumor, identifying residual early-stage T1 and T2 lesions, and evaluating small lymph nodes [12]. A meta-analysis revealed variable and inconsistent accuracy in addition to poor sensitivity (50%) of MRI in restaging after neoadjuvant therapy [13]. First, we found that the surgical plan and/or treatment plan was not altered in any patient by post-CRT MRI. Second, in our study, post-CRT MRI, in its current form when performed at a mean of 4.5 ± 2.2 weeks from the end of neoadjuvant therapy, had poor predictive value and was no better at predicting positive resection margins than pre-CRT MRI. Similarly, post-CRT MRI, in its current form, was not significantly more accurate in assessing T and N stage than pre-CRT MRI, highlighting the potential impact of post-radiation scarring and edema in decreasing the accuracy of post-CRT MRI. Third, interobserver variability among radiologists in terms of T and N restaging may be substantial [14]. Our data were consistent with previous findings, with staging modified by dedicated radiologists after the tumor board review in 20% of patients, further highlighting the utility of multidisciplinary tumor board review. Because prognosis depends on the pathologic stage rather than the clinical stage [15], and select patients may undergo nonoperative therapy (definitive CRT, “watch and wait”), the ability to predict the pathologic stage is crucial for decision-making. However, due to these restaging inaccuracies and interobserver variability, many surgeons still offer proctectomy to patients following CRT regardless of post-CRT MRI findings [10-14,16,17]. This is a reasonable treatment strategy, as even in experienced hands, five-year disease-free survival is only 57% in highly select patients in the “watch and wait” group in whom the recommendation for nonoperative therapy was based in part upon post-CRT MRI findings [18].

Our study has several limitations, including its retrospective approach, relatively small sample size, and single-center experience. Moreover, the study included patients over a five-year timeframe with varying MRI sequence techniques and nonhomogenous reporting. We attempted to minimize this effect by having dedicated radiologists re-review MRIs in a templated manner. Our study also excluded patients who underwent total neoadjuvant therapy, an increasingly common practice pattern. Another important consideration is the timing of post-CRT MRI, which was performed at a mean of 4.5 ± 2.2 weeks after neoadjuvant therapy in our study. While longer intervals may yield different results and may be more useful in determining complete clinical response, this interval was chosen for pragmatic reasons related to reviewing their cases in tumor board meetings preoperatively and having them scheduled for surgery in the appropriate time frame. Additionally, our data could not determine whether post-CRT MRI could reliably identify patients who would be able to undergo the “watch and wait” or organ-preserving approach [19-21]. We did not have any complete clinical responders, which may be due to the underlying patient population or the fact that the post-CRT MRI was performed at a mean of 4.5 ± 2.2 weeks following neoadjuvant therapy.

In summary, standard anatomic pulse sequences with imaging gestalt appear to be of limited use in the post-CRT setting, other than demonstrating disease progression which is rare. In particular, current understanding prohibits the accurate assessment of tumor viability. The current investigation into T2 signal characteristics [22] and diffusion-weighting imaging may increase future diagnostic performance of MRI in assessing tumor necrosis.

## Conclusions

Due to poor concordance with pathologic staging, inability to more accurately predict positive surgical margins than pre-CRT MRI, and the absence of a demonstrable impact on surgical treatment, the routine use of post-CRT restaging with MRI, in its current form when performed at the time interval used in our study, appears to be of limited clinical utility. A multidisciplinary approach to rectal cancer therapy appears to hold a substantial benefit.
